# Anand Model and Finite Element Analysis of Sn-0.3Ag-0.7Cu-3Bi Lead-Free Solder Joints in BGA Packages

**DOI:** 10.3390/ma19030636

**Published:** 2026-02-06

**Authors:** Junchen Liu, Abdullah Aziz Saad, Yuezong Zheng, Hongchao Ji, Zuraihana Bachok

**Affiliations:** 1 School of Mechanical Engineering, Universiti Sains Malaysia, Nibong Tebal 14300, Penang, Malaysia; junchen@ncst.edu.cn (J.L.); zuraihana@usm.my (Z.B.); 2 College of Mechanical Engineering, North China University of Science and Technology, Tangshan 063210, China; 3 School of Mechanical and Energy Engineering, Zhejiang University of Science and Technology, Hangzhou 310023, China

**Keywords:** Anand model, creep, finite element analysis, solder joints, Ball Grid Array packages

## Abstract

Bi-doped low-silver Sn-Ag-Cu solders are increasingly gaining attention in advanced electronic packaging due to their cost-effectiveness and enhanced mechanical properties. However, the thermo-mechanical reliability mechanisms of such modified solders, particularly Sn-0.3Ag-0.7Cu-3Bi (SAC0307-3Bi) within Ball Grid Array (BGA) assemblies, remain insufficiently understood. To address this gap, this research proposes a comprehensive assessment framework integrating constitutive parameter calibration with finite element analysis (FEA) to accurately characterize the mechanical behavior and fatigue durability of SAC0307-3Bi solder joints under cyclic thermal loads. The Anand viscoplastic parameters were first calibrated via the Norton creep law and virtual tensile tests. Subsequently, a 3D quarter-symmetry model was constructed to replicate thermal cycling conditions between 25 °C and 125 °C. Simulation data reveal a strong correlation between stress concentration and the Distance to Neutral Point (DNP), pinpointing the chip-side interface of the corner joint as the critical failure site. Moreover, creep strain was observed to accrue in a “step-wise” pattern, predominantly during the heating and cooling ramps, reflecting distinct temperature sensitivity. Utilizing the Syed model, the fatigue life was estimated at approximately 2239 cycles. These insights serve as a crucial benchmark for designing robust packages using Bi-doped, low-silver lead-free solders.

## 1. Introduction

**Figure 1:**
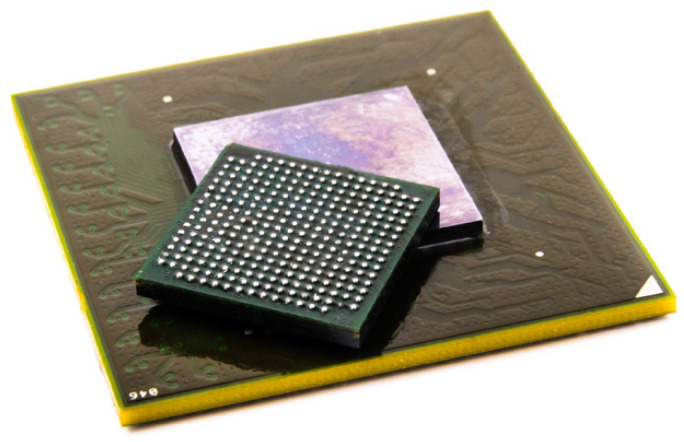
Ball Grid Array packages [5].

As illustrated in Figure 1, Ball Grid Array (BGA) packaging utilizes a regularly arranged array of solder balls to provide electrical interconnection and mechanical support. Characterized by high interconnect density, superior electrical performance, and high reliability, BGA technology has been widely adopted in modern electronic devices and has evolved rapidly to meet the growing demands for miniaturization and high integration [1,2,3]. In BGA assemblies, solder joints not only serve as electrical interconnects between the chip and the Printed Circuit Board (PCB) but also play a crucial role in accommodating the deformation caused by the thermal expansion mismatch among different packaging materials. However, electronic packages are typically subjected to complex and varying environmental loads during service, with temperature fluctuations having the most significant impact [4].

Due to the significant mismatch in coefficients of thermal expansion (CTE) among the silicon chip, substrate, solder joints, and PCB, periodic shear deformation and multiaxial stress concentration are induced under thermal cycling loads. As critical components providing both electrical and mechanical connections, solder joints are highly susceptible to stress concentration, creep deformation, and damage accumulation during this thermo-mechanical coupling process [6,7]. Consequently, they become the most vulnerable sites within the BGA package structure. Therefore, the thermo-mechanical reliability of solder joints has emerged as a critical bottleneck limiting the long-term service performance of BGA devices.

Driven by environmental regulations and legislative mandates, the electronic packaging industry has progressively transitioned to lead-free interconnects, with the Sn–Ag–Cu (SAC) series becoming the most widely adopted solution [8]. Among these, low-Ag SAC solders have garnered significant attention due to their cost-effectiveness and favorable processability. However, reducing the Ag content inevitably compromises the mechanical properties and creep resistance of the solder, thereby adversely affecting the long-term reliability of solder joints [9,10]. To mitigate these deficiencies, micro-alloying has emerged as a critical strategy to enhance the comprehensive performance of low-Ag solders [11]. Among various potential alloying elements, Bismuth (Bi) has been identified as a superior candidate due to its remarkable strengthening efficiency. Functioning as an ideal cost-effective alternative to high Ag content, Bi atoms induce significant solid solution strengthening within the β-Sn matrix, thereby effectively compensating for the mechanical deficiencies of low-Ag solders. Consequently, Bi is regarded as a highly promising modifier, demonstrating superior potential for enhancing the reliability of SAC interconnects compared to other conventional dopants [12,13].

Finite element numerical simulation has emerged as a vital instrument for assessing the reliability of lead-free solder BGA packages under thermal cycling loads, serving as a key means to elucidate the mechanical response, thermal durability, and failure mechanisms of solder joints [6,14,15]. Existing research demonstrates that the incompatible deformation, induced by the mismatch in coefficients of thermal expansion (CTE) among packaging components, subjects solder joints to significant shear stress and creep damage during thermal cycling. Furthermore, the characteristics of stress distribution, inelastic strain accumulation, and damage evolution are intimately tied to the package structure design and the constitutive properties of the solder [1,16]. Building on this understanding, researchers have achieved quantitative assessments of solder joint thermal fatigue life by integrating key mechanical parameters extracted from finite element analysis (such as stress, creep strain, or creep energy density) with fatigue life prediction models [17,18]. However, despite the extensive application of the aforementioned methodologies in the reliability analysis of conventional SAC solder systems and other alloy-modified solders, systematic and in-depth investigation into Bi-modified low-silver SAC solder BGA packages—particularly regarding their stress–creep interaction behavior and life evolution laws under complex thermo-mechanical coupling conditions—remains lacking.

Based on this background, this paper investigates the thermo-mechanical reliability of the BGA package using Sn-0.3Ag-0.7Cu-3Bi (SAC0307-3Bi) lead-free solder. The specific research objectives and the optimized parameters are outlined as follows: 1. Constitutive Parameter Optimization: By integrating the Norton creep equation with virtual tensile simulations, the nine key parameters of the Anand viscoplastic constitutive model (specifically A, Q/R, m, ξ, h_0_, a, $\hat{s}$, n and s_0_) were calibrated and optimized for the SAC0307-3Bi solder. 2. Stress–Strain Evolution Analysis: A 3D quarter-symmetric finite element model was constructed to simulate the spatiotemporal evolution of stress, creep strain, and damage accumulation under thermal cycling conditions (25 °C to 125 °C). 3. Fatigue Life Prediction: Based on the stabilized creep behavior, the thermal fatigue life of the modified solder joints was quantitatively assessed using the Syed life prediction model. The findings of this research aim to provide a theoretical basis and data support for the reliability design of low-Ag, Bi-containing lead-free solders in advanced packaging applications.

## 2. Anand Model and Material Properties

Under the thermal cycling loads associated with BGA packaging, solder joints typically operate within a high homologous temperature regime ($T/T_{m}>0.5$). Consequently, their mechanical response exhibits pronounced time-dependent and nonlinear characteristics, wherein creep deformation plays a dominant role in the process of damage evolution. To accurately simulate how solder joints react during thermal cycling, it is crucial to use a constitutive model that captures their viscoplastic behavior. Specifically, the model must account for the interaction between stress, strain rate, and temperature.

The Anand constitutive model is a unified viscoplastic model initially developed for high-temperature metal forming operations. Unlike classical plasticity theories, it does not require an explicit yield surface or loading/unloading criteria. Instead, it employs a single scalar internal variable, Տ (representing isotropic deformation resistance), to capture the material’s microstructural evolution, such as dislocation density and grain size effects. The formulation of the Anand model is primarily governed by two coupled equations: the flow equation and the evolution equation. The flow equation relates the inelastic strain rate to the equivalent stress, temperature, and deformation resistance. The evolution equation describes how the deformation resistance Տ evolves through the competition between strain hardening and dynamic recovery processes. This makes the model particularly suitable for simulating the creep and stress relaxation behavior of solder alloys under thermal cycling conditions [19].

Numerous studies have proven that the Anand model is highly accurate in predicting stress, creep, and fatigue in lead-free solder joints during thermal cycling. As a result, it is widely used for reliability analysis in packaging structures such as BGA, Chip Scale Package (CSP), and Flip Chip (FC). For instance, Long et al. [20] verified that the Anand model effectively captures the flow characteristics of SAC solders across a wide range of temperatures and strain rates. Recently, Hu et al. [15] used this model to simulate stress in BGA packages. They also calculated how internal defects in the solder joints affect their fatigue life. Given that the Anand model has been fully validated and widely recognized for describing the viscoplastic deformation characteristics of lead-free solders, this study employs the Anand viscoplastic constitutive model to describe the mechanical behavior of SAC0307-3Bi solder joints. The corresponding flow equation and evolution equations are expressed as follows [21].

Anand model deformation impedance is proportional to the equivalent stress

(1) σ=cs; c < 1

where s denotes the internal state variable, and c is a material parameter. Under the condition of a constant strain rate, c remains constant. The expression is given by:

(2) $$c=\frac{1}{\xi }\sin h^{-1}\left\{{\left[\frac{{\dot{\varepsilon }}_{p}}{A}\exp\left(\frac{Q}{RT}\right)\right]}^{m}\right\}$$

where:

ξ represents the stress coefficient;

R is the universal gas constant;

${\dot{\varepsilon }}_{p}$ denotes the inelastic strain rate;

A is the pre-exponential factor; 

m is the strain rate sensitivity;

T is the absolute temperature;

Q is the activation energy. 

The Anand model defines the flow equation as a function of the internal state variable s and temperature T, and the specific expression is given by:

(3) ε˙p=Aexp(−QRT)[sinh(ξσs)]1m

The expression for s is:

(4) $$\dot{s}=h\left(\sigma ,s,T\right){\dot{\varepsilon }}_{p}$$

where h(σ,s,T) represents the evolution equation of the internal state variable, which characterizes the competition between dynamic strain hardening and dynamic recovery processes. The specific expression derived from the Anand model is as follows:

(5) $$\dot{s}=\left[h_{0}{\left(1-\frac{s}{s^{*}}\right)}^{a}\mathrm{sign}\left(1-\frac{s}{s^{*}}\right)\right]{\dot{\varepsilon }}_{p};a>1$$

(6) $$s^{*}=\hat{s}{\left[\frac{{\dot{\varepsilon }}_{p}}{A}\exp\left(\frac{Q}{RT}\right)\right]}^{n}$$

By combining Equations (1), (2) and (6), the expression for the saturation stress $\sigma^{*}$ under a given temperature and strain rate is derived as follows:

(7) $$\sigma^{*}=cs^{*}=\frac{\hat{s}}{\xi }{\left[\frac{{\dot{\varepsilon }}_{p}}{A}\exp\left(\frac{Q}{RT}\right)\right]}^{n}\sin h^{-1}\left\{{\left[\frac{{\dot{\varepsilon }}_{p}}{A}\exp\left(\frac{Q}{RT}\right)\right]}^{m}\right\}$$

Equation (8) is derived by combining Equations (1) and (5) taking the partial derivative with respect to the inelastic strain rate ${\dot{\varepsilon }}_{p}$.

(8) $$\frac{d\sigma }{d{\dot{\varepsilon }}_{p}}=ch_{0}{\left|1-\frac{\sigma }{\sigma^{*}}\right|}^{a}\mathrm{sign}\left(1-\frac{\sigma }{\sigma^{*}}\right);>1$$

The integral of Equation (8) gives Equation (9):

(9) σ=σ∗−{(σ∗−cs0)(1−a)+(a−1)[ch0(σ∗)(−a)]ε˙p}1(1−a)

where:

s* is the saturation value of deformation resistance dependent on temperature and strain rate;

h_0_ is the hardening constant;

a is the strain rate sensitivity of hardening;

$\hat{s}$ is the coefficient for deformation resistance saturation value;

n is the strain rate sensitivity of the saturation value;

s_0_ is the initial value of deformation resistance.

The nine parameters characterizing the Anand viscoplastic model (A, Q, m, ξ, h_0_, a, $\hat{s}$, n and s_0_) can be derived by fitting data obtained from constant strain rate virtual tensile simulations. The sequential procedure for calculating these parameters is as follows:

1. Six parameters, A, $\hat{s}$, m, ξ, n and Q/R, are obtained by fitting Equation (7) to the saturation stress (UTS) data. This is done using data from different strain rates and temperatures.
2. The other three parameters (s_0_, a, and h_0_) are calculated by fitting Equation (9) to the stress versus plastic strain data under various conditions [22].

**Figure 2:**
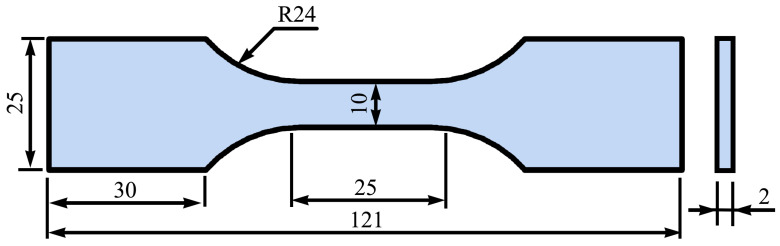
Constant strain rate tensile specimen size (Unit: mm).

Based on the Norton creep constitutive equation for SAC0307-3Bi solder determined in a previous study, the theoretical creep stress values under various strain rates were initially calculated. Subsequently, virtual tensile simulations were conducted using a finite element model with geometric dimensions identical to the experimental specimens, as illustrated in Figure 2. Numerical simulations were conducted to generate stress–strain curves across a comprehensive range of temperature and strain rate combinations. The specific testing conditions are summarized in Table 1.

(10) $$\varepsilon =3.82\sigma^{4.37}\exp\left(-\frac{64.43}{\mathrm{RT}}\right)$$

For the baseline Sn-0.3Ag-0.7Cu solder [23], the material properties are defined as follows: CTE of 2.31 × 10^−5^ K^−1^, Young’s modulus of 47,500 MPa, density of 7.33 g/cm^3^ and Poisson’s ratio of 0.30. Regarding the SAC0307-3Bi solder, the overall stiffness of the alloy is enhanced compared to the traditional SAC0307, primarily attributed to the solid solution strengthening effect of Bi atoms in the Sn matrix and their refining effect on the microstructure. Based on mechanical property test data from relevant literature, the Poisson’s ratio and Young’s modulus of the solder were set to 0.30 and 50,000 MPa, respectively, in this study [24,25]. Furthermore, research indicates that the addition of Bi can effectively suppress the thermal expansion behavior of SAC solders. Accordingly, the CTE of SAC0307-3Bi in this model was set to 2.0 × 10^−5^ K^−1^ [13] to accurately capture the thermal deformation characteristics of the material.

**Figure 3:**
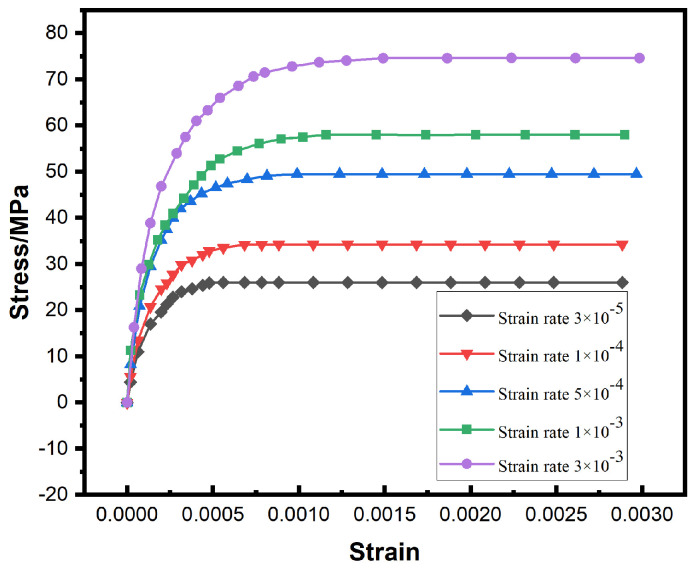
Stress–strain relationship of specimens at different strain rates at 25 °C.

Figure 3 presents the stress–strain response curves of the SAC0307-3Bi solder at 25 °C under various strain rates. The results show that the stress increases sharply with strain during the initial deformation stage, then rapidly stabilizes and maintains a saturation level, exhibiting significant steady-state flow characteristics. This stress evolution generally conforms to typical viscoplastic deformation laws, revealing that the material is primarily governed by time-dependent steady-state creep mechanisms after yielding. However, minor discrepancies exist between the simulated response and theoretical data. These deviations are primarily attributed to the homogenization assumption of the model, which treats the solder as an isotropic and homogeneous continuum, thereby inevitably simplifying the complex localized interactions between the β-Sn matrix and Intermetallic Compounds found in actual experimental measurements.

**Figure 4:**
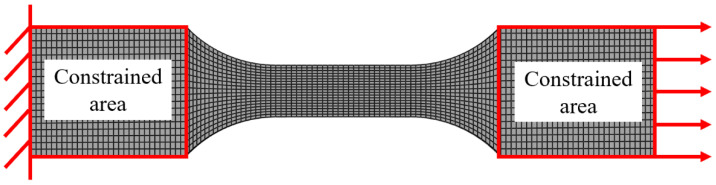
Boundary conditions of simulation.

Building upon the constitutive relationship established via the Norton equation, virtual tensile simulations under constant strain rates were further conducted. The geometry and boundary conditions of the finite element (FE) model are illustrated in Figure 4. During the material definition phase, the derived stress–strain data were incorporated into the model. Additionally, a ductile damage criterion with a fracture threshold set to 30% was introduced to characterize the failure behavior of the material.

**Figure 5:**
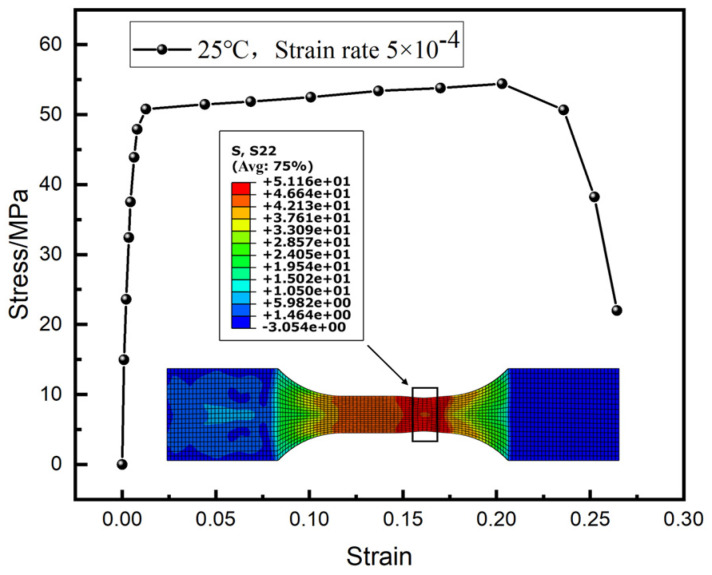
Simulation results at 25 °C and strain rate of 5.0 × 10^−4^.

Taking the typical loading condition at 25 °C with a strain rate of 5.0 × 10^−4^ s^−1^ as an example, the simulation results are illustrated in Figure 5. The results indicate that the stress climbs sharply to 51.48 MPa during the initial loading stage, subsequently entering a steady-state flow phase and gradually approaching a saturation value of approximately 54.43 MPa. Thereafter, influenced by stress softening induced by accumulated damage, the stress curve drops rapidly, leading to specimen fracture. The critical equivalent strain prior to fracture, as determined by the simulation, is approximately 26.43%.

The saturation stress values of the material obtained through simulation under various temperature and strain rate conditions are summarized in Table 2. It is evident that at a constant strain rate, the saturation stress decreases with increasing temperature, exhibiting a distinct negative correlation. Conversely, under constant temperature conditions, the saturation stress increases correspondingly with the increase in strain rate. These variation trends are consistent with the laws observed in creep experiments, indicating that the simulation results can accurately reflect the actual mechanical response of the material under different thermo-mechanical loading conditions.

The physical mechanism governing these variations lies in the competition between strain hardening and dynamic recovery. At elevated temperatures, the thermal activation energy facilitates dislocation climb and glide, thereby accelerating the dynamic recovery process and leading to a significant reduction in saturation stress. Conversely, higher strain rates induce rapid dislocation multiplication, which dominates over the recovery rate, resulting in increased deformation resistance. This distinct sensitivity to both temperature and strain rate confirms that the SAC0307-3Bi solder exhibits typical viscoplastic characteristics suitable for description by the Anand model.

Finally, through systematic fitting of the simulation results, the nine key material parameters of the Anand viscoplastic constitutive model for the SAC0307-3Bi solder were determined; their specific values are listed in Table 3. It is noteworthy that Zheng et al. [23] previously validated the effectiveness of this “virtual tensile-parameter inversion” method for the SAC0307 solder system. Their findings demonstrated that the relative error between the constitutive parameters obtained via inversion and the experimentally measured values was approximately 2%. Given the proven computational accuracy of this methodology, the Anand constitutive parameters obtained for the SAC0307-3Bi solder in this study are considered highly reliable and serve as valid inputs for describing the material behavior of solder joints in the subsequent finite element analysis.

The derived Anand parameters quantitatively reflect the microstructural strengthening effects of the Bismuth addition. Specifically, the initial value of deformation resistance (S_0_ = 26.09 MPa) and the hardening constant (h_0_ = 8850 MPa) are relatively high. This can be attributed to the solid solution strengthening effect of Bi atoms, which dissolve in the Sn matrix and effectively impede dislocation movement. These parameters collectively characterize the enhanced mechanical stiffness and creep resistance of the SAC0307-3Bi alloy compared to standard lead-free solders.

## 3. Creep Simulation Under Thermal Cyclic Loading

Focusing on the SAC0307-3Bi lead-free solder BGA package, this study investigates the evolution of stress states and creep behavior under thermal cycling loads ranging from 25 °C to 125 °C. Given the significant mismatch in CTE among the molding compound, chip, solder joints, and PCB, severe incompatible deformation occurs among these components during thermal cycling. This, in turn, subjects the solder joints to high-intensity interfacial shear stress. Extensive research indicates that under cyclic thermal loading, this shear stress serves as the core mechanism driving creep deformation and dominating the evolution of damage accumulation in solder joints [1,26,27].

To optimize computational efficiency while ensuring the accuracy of the stress–strain response calculation, necessary and rational simplifications were applied to the finite element model:

(1) Micron-scale intermetallic compounds (IMCs) and minute plating structures at the solder interface were neglected to focus on the simulation of macroscopic creep behavior;
(2) Given the small physical dimensions of the package, transient heat conduction and thermal convection effects were ignored, and the entire model was assumed to be in a uniform temperature field during any given load step.

**Figure 6:**
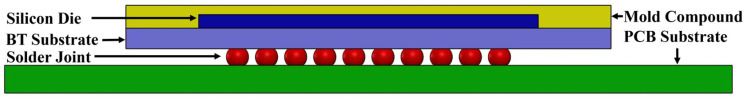
Cross-section of simplified BGA package.

Based on the aforementioned assumptions, a three-dimensional (3D) finite element model, as illustrated in Figure 6, was constructed using a standard BGA package from TOPLINE Corporation as the prototype. The model comprises five primary components: the silicon chip, BT substrate, molding compound, PCB, and SAC0307-3Bi lead-free solder joints. The detailed geometric parameters for each component are presented in Table 4.

In the finite element model, the PCB, BT substrate, Si chip, and Epoxy Molding Compound (EMC) were assumed to be isotropic linear elastic materials. In contrast, due to the low-melting-point nature of SAC series lead-free solders, their homologous temperature under service conditions typically exceeds 0.5, thereby exhibiting pronounced time-dependent viscoplastic characteristics. Under thermal cycling, solder joints suffer from severe inelastic deformation. To address this, the Visco quasi-static method in Abaqus 2022 (Dassault Systèmes, Vélizy-Villacoublay, France) was used to simulate this creep behavior. Additionally, the Anand model was applied to describe the mechanical response of the SAC0307-3Bi solder under mechanical and thermal loads. The material parameters used for this simulation are listed in Table 5.

While the finite element model and life prediction methodology presented in this study offer valuable insights into the creep–fatigue behavior of SAC0307–3Bi solder joints, several limitations arising from the simplified assumptions must be acknowledged.

First, concerning material properties, the PCB and BT substrate were modeled as isotropic linear elastic materials. In reality, these components, as composite laminates, exhibit significant orthotropy, particularly possessing a higher coefficient of thermal expansion (CTE) in the thickness direction (Z-axis). This simplification might, to some extent, underestimate the deformation mismatch in the vertical direction.

Second, the model neglects microstructural evolution, specifically the formation and growth of intermetallic compounds (IMCs) at the solder interface. Since an excessive IMC layer can induce brittle fracture—a failure mode distinct from creep–fatigue—the current model may not fully capture failure mechanisms dominated by interfacial embrittlement in the later stages of cycling.

Third, the analysis assumes a uniform temperature field, ignoring transient heat conduction effects. In actual service conditions, transient temperature gradients between the chip and substrate during rapid heating or cooling processes could induce additional thermal stresses, which were not considered in this study.

Finally, due to the absence of specific fatigue data for Bi-containing alloys, the constants for the Syed life prediction model were adopted from standard SAC solder data. Although the enhancement of creep resistance by Bi is captured through the Anand parameters, its potential impact on fatigue ductility was not explicitly modeled. Consequently, the fatigue life predicted in this paper should be regarded as a comparative benchmark rather than as absolute values.

**Figure 7:**
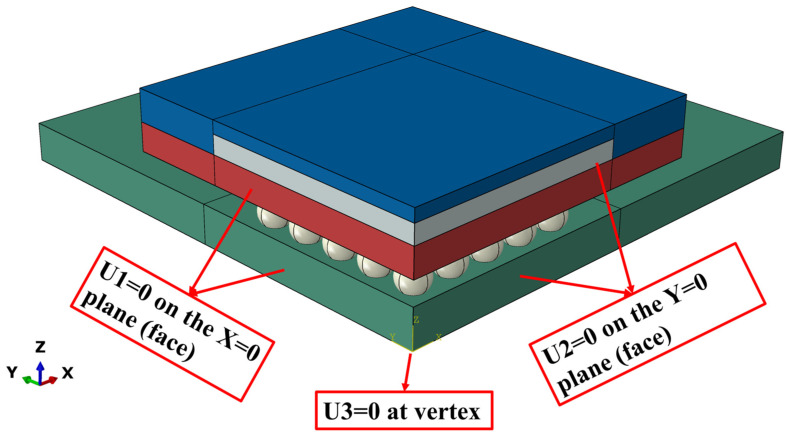
Quarter-symmetric model of the BGA assembly, and boundary conditions.

As illustrated in Figure 7, given the significant geometric and thermal load symmetry of the BGA package structure, a three-dimensional (3D) quarter-symmetric finite element model containing the full solder array was established to maximize computational efficiency while ensuring accuracy. To clarify the physical mechanisms and ensure simulation reproducibility, the following key assumptions were adopted during the modeling process:

(1) Material Definition: The solder alloy is defined as a viscoplastic material, while other components (chip, substrate, and PCB) are simplified as linear elastic, isotropic, and homogeneous materials.
(2) Contact Interfaces: The interfaces between components are assumed to be “perfectly bonded,” ignoring the effects of delamination or relative sliding.
(3) Initial State: The model is assumed to be in a stress-free state at the initial moment of the simulation.

On this basis, to accurately simulate real mechanical constraints, strict symmetry boundary conditions were applied to the symmetry planes: specifically, the displacement in the X-direction (U1 = 0) was constrained on the X = 0 plane, and the displacement in the Y-direction (U2 = 0) was constrained on the Y = 0 plane. Furthermore, to eliminate rigid body motion in the Z-axis (thickness direction) without introducing over-constraints, a single point constraint (U3 = 0) was applied to the center node at the bottom of the PCB, located at the intersection of the two symmetry planes. These settings allow for free thermal expansion in unconstrained directions, consistent with actual physical operating conditions.

A non-uniform meshing strategy was applied to the model. This ensures accurate results while maintaining high calculation efficiency. Considering that under thermal cycling loads, stress concentrations and inelastic creep deformations are primarily located at the outermost solder joints (which have the maximum distance from the neutral point) and the region directly beneath the chip edge, local mesh refinement was applied to these critical regions to accurately capture the local severe stress gradients.

**Figure 8:**
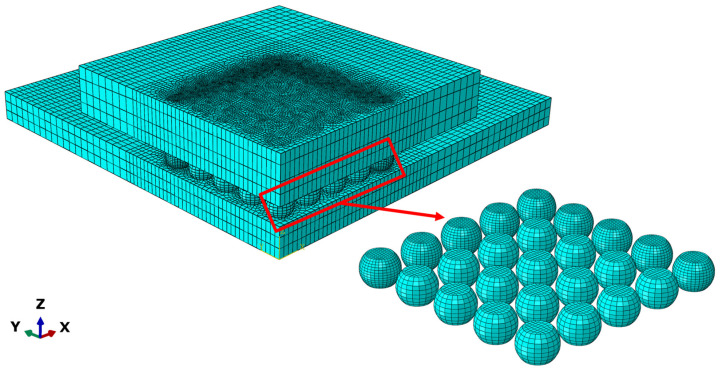
Meshing of Quarter-symmetric model of the BGA assembly.

C3D8R elements were used for the entire model. These elements are 8-node linear hexahedral types with reduced integration. This element type is known for exhibiting excellent convergence performance when handling large deformation and contact problems. The finally established finite element model comprises a total of 76,384 elements, with specific meshing details illustrated in Figure 8.

**Figure 9:**
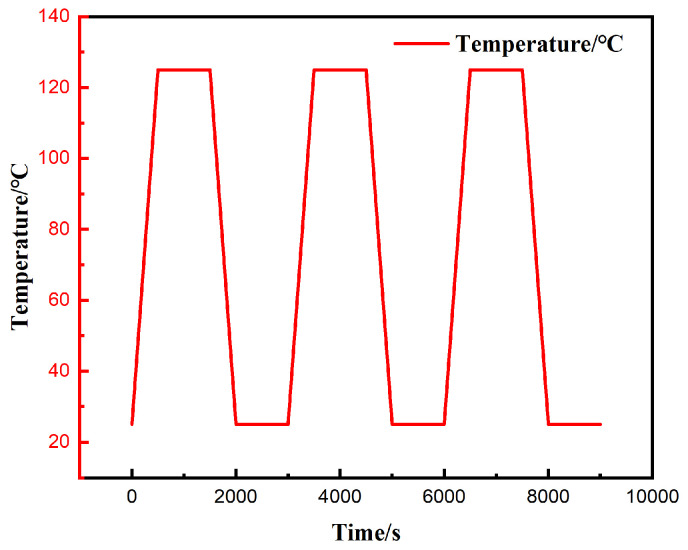
Thermal cycling profile with 3 cycles.

To ensure that the simulation results are comparable with the solder creep experiments, the thermal cycling load parameters for the finite element analysis were established based on the SAC0307-3Bi material testing conditions. The specific temperature–time loading profile is presented in Figure 9. The cycling temperature range covered 25 °C to 125 °C, and the initial ambient temperature was set to 25 °C.

Specifically, the ramp rates for both the heating and cooling phases were controlled at 0.2 °C/s, corresponding to a duration of 500 s for each ramping process. The dwell times for both the low-temperature stages and high-temperature were set to 1000 s; consequently, the duration of a single complete thermal cycle was 3000 s. Given that the stress–strain hysteresis response of low-melting-point solders typically requires several cycles to achieve closure and stabilization, three consecutive thermal cycling loads were applied to the model as shown in Figure 8, resulting in a total analysis time of 9000 s. This approach allows for the extraction of creep response characteristics of the solder joints during the stable stage.

## 4. Results and Discussion

**Figure 10:**
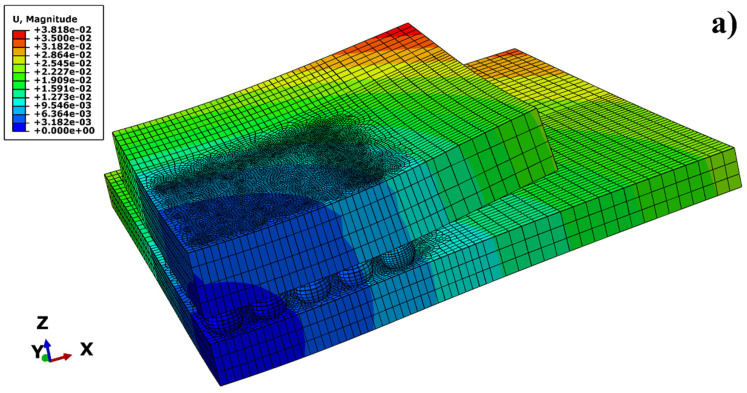

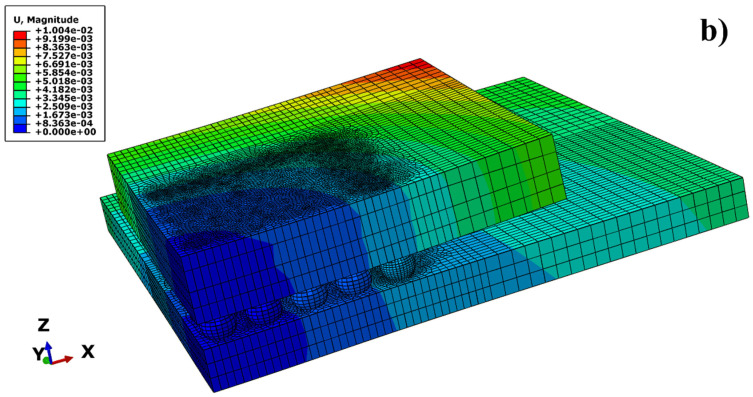
Warpage during the insulation stage of the BGA model (scaling factor: 1:100): (**a**) High temperature; (**b**) Low temperature.

Figure 10 illustrates the global displacement contour plots of the model during the low-temperature and high-temperature dwell stages. It can be observed from the figure that the model exhibits pronounced concave warpage during the high-temperature dwell stage. In contrast, during the low-temperature dwell stage, the model manifests only slight convex warpage. A comparison between the two states reveals that the magnitude of warpage during the low-temperature stage is significantly smaller than that during the high-temperature stage, indicating a lesser degree of overall deformation.

The mechanism analysis for this phenomenon is as follows: The temperature cycling range established in this study is 25 °C to 125 °C, with the initial temperature of the model set at 25 °C. Furthermore, residual stresses typically introduced during the reflow cooling process were not preset. Therefore, the low-temperature dwell stage (25 °C) is in the vicinity of the reference temperature of the model. Consequently, the thermal strain induced by the mismatch of CTE is minimal, resulting in insignificant global warpage (the slight warpage observed at this stage is primarily attributed to the inelastic deformation accumulated during the high-temperature stage).

In sharp contrast, during the high-temperature dwell stage (125 °C), due to the significant disparity in CTE among the chip, substrate, and solder components, the substantial temperature rise triggers distinct thermal expansion mismatch effects. This intense thermo-mechanical coupling interaction leads to substantial bending deformation of the model, macroscopically manifesting as pronounced concave warpage.

**Figure 11:**
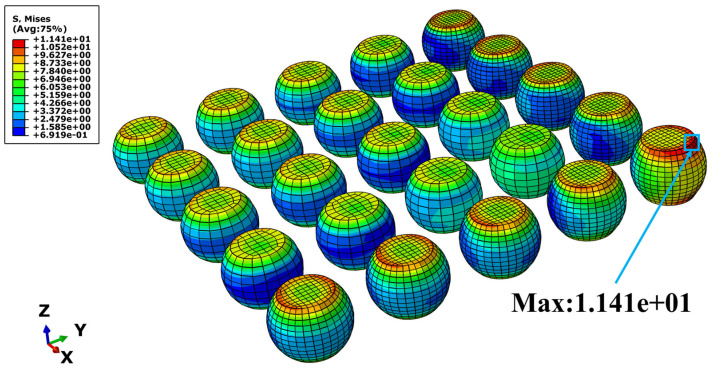
Mises equivalent stress cloud map.

As illustrated in Figure 11, the contour plot reveals the distribution of von Mises equivalent stress across the solder joints. The peak stress value reaches 11.41 MPa and is concentrated at the upper interface of the outermost corner joint. This finding confirms that thermal cycling induces significant stress localization, particularly at the corner joints and their interfacial regions.

Since the solder joints at the four corners of the PCB are subjected to the largest thermal expansion mismatch deformation within the structure, their upper and lower surfaces are more susceptible to higher equivalent stress levels under cyclic thermal loading. Under the long-term repeated action of high stress, these regions serve as potential critical locations for internal damage accumulation and crack initiation. With the continuous accumulation of stress and creep damage, the failure risk of corner solder joints is significantly higher than that of the internal joints, thereby potentially compromising the overall reliability of the packaging structure.

To characterize the stress evolution patterns of BGA solder joints under thermal cycling loads, this study comprehensively selected von Mises equivalent stress, longitudinal shear stresses (S13, S23), and Tresca stress as analysis metrics. The von Mises stress serves to macroscopically locate regions of stress concentration. Meanwhile, considering that longitudinal shear induced by CTE mismatch constitutes the dominant mechanism leading to creep–fatigue failure in solder joints, the S13 and S23 components are utilized to more accurately reflect the physical nature of the damage. Additionally, the Tresca stress, serving as an indicator of maximum shear stress, effectively supplements the characterization of stress states under extreme loading conditions. By selecting the high-temperature and low-temperature dwell stages as characteristic time points, this study compares the distributional evolution of these three stress indicators, thereby elucidating the differences in mechanical response between the creep-dominated and residual-stress-dominated stages.

**Figure 12:**
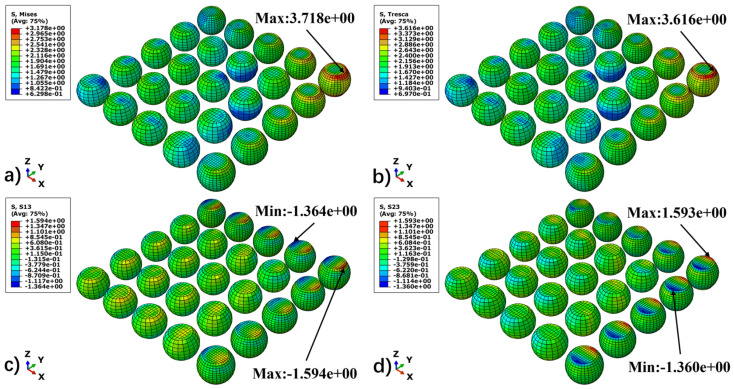
Stress distribution of the solder joint at the 125 °C dwell stage. (**a**) Mises, (**b**) Tresca, (**c**) S13, and (**d**) S23.

**Figure 13:**
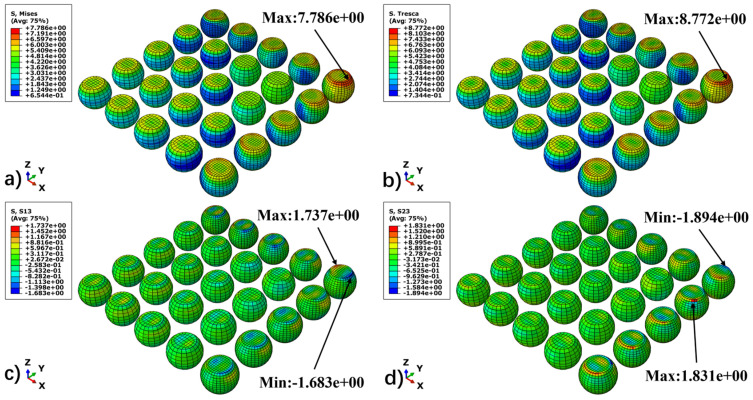
Stress distribution of the solder joint at the 25 °C dwell stage. (**a**) Mises, (**b**) Tresca, (**c**) S13, and (**d**) S23.

Figure 12 and Figure 13 illustrate the stress field distributions of the BGA solder joint array at the end of the high-temperature and low-temperature dwell stages, respectively. Through a comparative analysis, several key mechanical characteristics can be identified.

First, the stress distribution of the solder joint array exhibits a distinct dependence on the distance from the neutral point (DNP) [29]. Both the von Mises and Tresca equivalent stresses display a radial gradient, increasing from the center of the array toward the periphery, with the peak stress consistently located at the outermost corner solder joint along the diagonal. Furthermore, high-stress regions are primarily concentrated at the interconnect interfaces between the solder and the BT substrate and PCB. This is attributed to the CTE mismatch among materials, which induces the maximum shear strain gradient at these interfaces.

Second, the stress levels differ significantly across different temperature stages, with the magnitude of residual stress at low temperatures being notably higher than that at high temperatures. The underlying physical mechanism for this phenomenon is as follows: during the high-temperature dwell stage (125 °C), the solder undergoes intense viscoplastic creep flow, allowing the accumulated thermal mismatch stress to be effectively relaxed and released. Conversely, during the low-temperature stage (25 °C), as the creep effect is significantly diminished, the stress relaxation mechanism is restricted, resulting in high residual stresses being retained within the solder joints.

Finally, the distribution of shear stress components confirms that multiaxial shear is the dominant loading mode for the solder joints. The S13 and S23 components dominate the thermal deformation shear components along the X-axis and Y-axis, respectively. Due to the vector superposition of these bidirectional shear stresses, the corner solder joint experiences the most severe stress state, rendering it the critical region most susceptible to fatigue damage initiation within the entire packaging structure.

**Figure 14:**
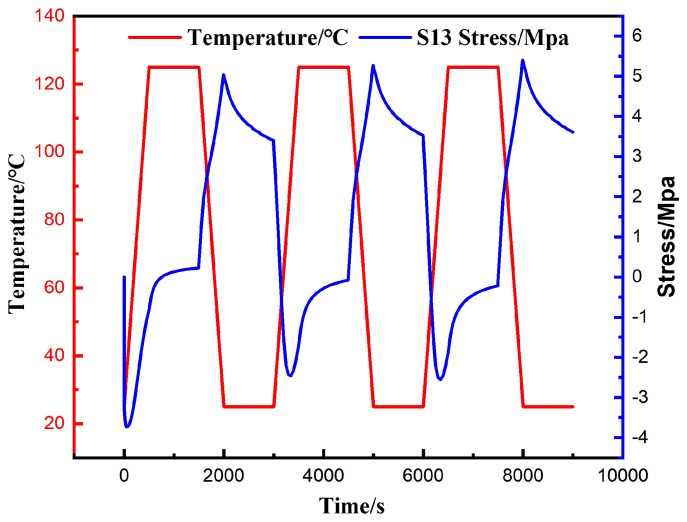
Curve of maximum S13 shear stress with thermal cycling.

**Figure 15:**
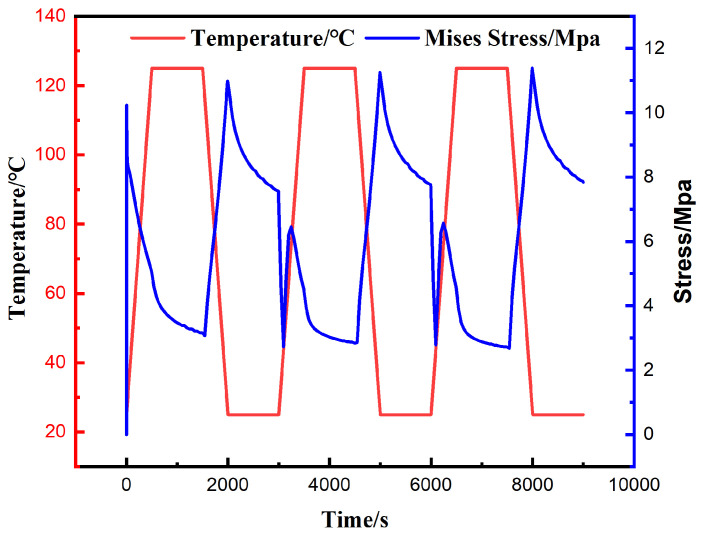
Curve of maximum Mises equivalent stress with thermal cycling.

The finite element analysis results indicate that the peak values of von Mises stress, Tresca stress, and the longitudinal shear stress components S13 and S23 all occur at the outermost corner solder joint of the array. In light of this observation, the critical element exhibiting the highest degree of stress concentration within this corner joint was selected as the focal point of this study to thoroughly investigate its local mechanical behavior. Figure 14 and Figure 15 clearly illustrate the evolution curves of the maximum equivalent stress and maximum shear stress of this critical element with respect to temperature, respectively.

Figure 14 depicts the time-dependent evolution curve of the shear stress experienced by the critical strain concentration element within the corner solder joint. The data indicate that the shear stress at this location exhibits distinct periodic alternating characteristics, with its value fluctuating cyclically between −3.73 MPa and 5.39 MPa. With the exception of the reverse shear stress amplitude in the first cycle being slightly higher than that in subsequent cycles, the overall cyclic response trend remains highly consistent.

The analysis reveals a clear correspondence between the distribution of shear stress extrema and the temperature load: the peak temperature moment corresponds to the maximum negative shear stress, while the valley temperature moment corresponds to the maximum positive shear stress. Although stress relaxation phenomena occurred during both the high-temperature and low-temperature dwell stages, the residual stress levels following relaxation remained relatively high. Based on this observation, it can be inferred that the dwell stages represent high-risk intervals for solder joint failure. During these stages, the combined effect of the sustained high stress state and the accumulated inelastic strain significantly accelerates crack initiation and propagation, thereby dominating the final fatigue failure of the solder joints.

Figure 15 illustrates the dynamic evolution curve of the von Mises equivalent stress for the critical element of the corner solder joint over time, with values fluctuating cyclically within the range of 0 MPa to 11.41 MPa. Overall, the stress response exhibits periodic variations highly correlated with the temperature load and stabilizes rapidly following the initial adjustment of the first cycle.

It is noteworthy that the stress unloading process during the heating stage does not follow a completely monotonic decay trend; instead, it is accompanied by brief, slight stress rebound fluctuations. Conversely, during the cooling stage, driven by the thermal contraction of the materials, the equivalent stress continues to accumulate and climb, reaching the global peak at the onset of the low-temperature dwell. Furthermore, significant stress relaxation phenomena are observed during both dwell stages.

In summary, given that low-temperature conditions are the critical factor subjecting solder joints to limit stress load; to enhance the fatigue life of the device, it is recommended to minimize the frequency and duration of equipment operation in extreme low-temperature environments in practical applications.

**Figure 16:**
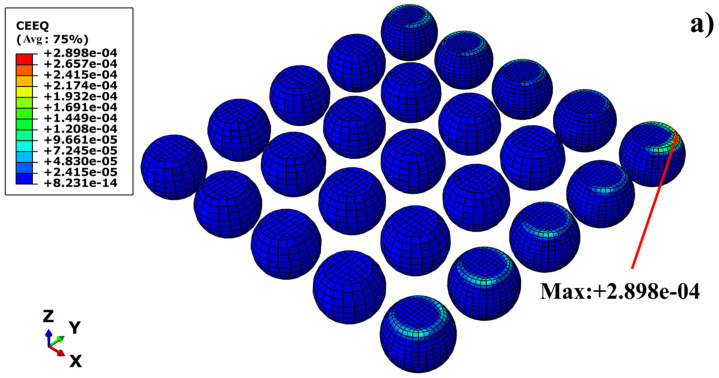

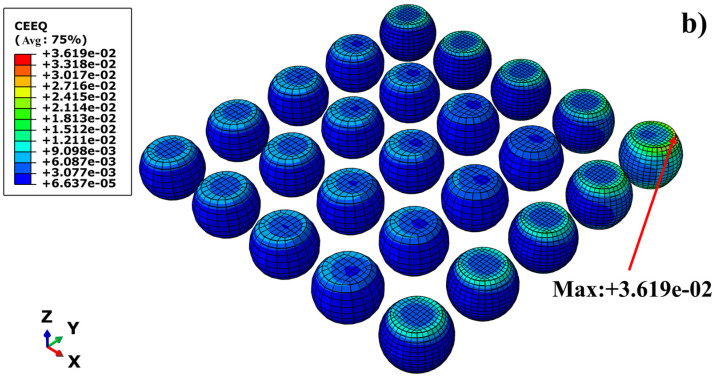
The equivalent creep strain of BGA solder joint under cyclic temperature load: (**a**) after the cycle begins, and (**b**) before the end of the cycle.

Given that creep deformation is the dominant mechanism leading to fatigue failure of solder joints under thermal cycling loads, this study focuses on examining the spatiotemporal evolution laws of solder joint creep behavior. Figure 16 illustrates the equivalent creep strain distribution contours of the solder joint array at the initial stage and final stage of cycling, respectively, to intuitively contrast the accumulation characteristics and distribution patterns of creep damage.

A comparison of Figure 16 reveals that, in both the initial and final stages of thermal cycling, regions of high equivalent creep strain are consistently concentrated at the contact interfaces between the chip/PCB and the outermost solder joints of the array. In particular, the interface region of the corner solder joint on the chip side exhibits the most significant creep accumulation. This distribution pattern maintains high consistency with the previously described equivalent stress distribution characteristics.

**Figure 17:**
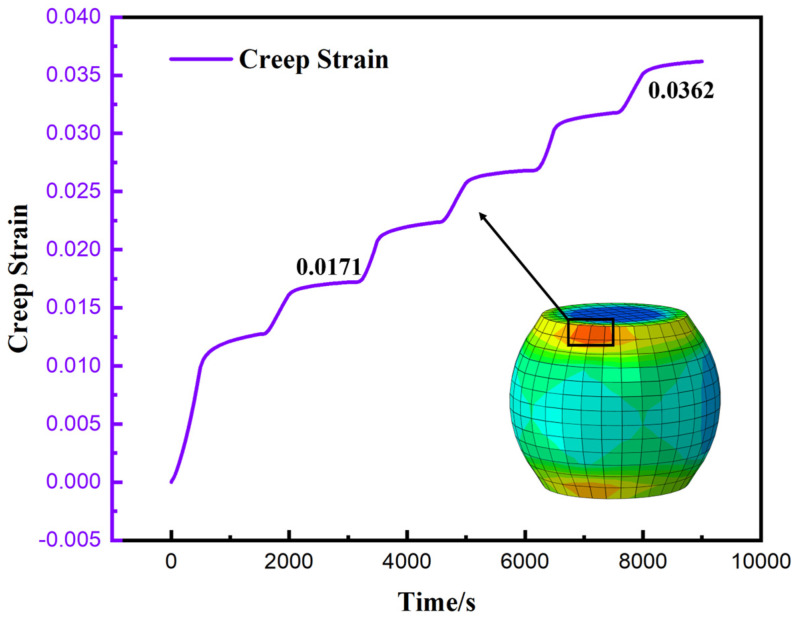
Historical output curve of maximum equivalent creep strain at corner weld joints.

Given that solder joint failure under thermal cycling loads is primarily attributed to the interaction between creep and fatigue, the region where the maximum creep strain occurs often indicates the potential path for crack initiation and propagation. Therefore, to quantitatively characterize the damage accumulation process in this critical region, Figure 17 extracts and presents the time-dependent evolution curve of the equivalent creep strain for the element exhibiting the maximum creep within the corner solder joint.

Figure 17 illustrates the cumulative evolution history of the equivalent creep strain for the critical element of the corner solder joint over time. The data indicate that the equivalent creep exhibits a typical “step-wise” growth characteristic. The cyclic shear stress, driven by the CTE mismatch, acts as the primary driving force for this inelastic deformation. Despite the high shear stress concentration at the corner joint, the increment of creep strain per cycle tends to stabilize rapidly. This indicates that the competition between strain hardening and dynamic recovery (governed by the Anand model) reaches a dynamic equilibrium, thereby ensuring the cyclic stability of the simulation results. Consequently, during the first thermal cycle, the material underwent the most significant inelastic deformation, with the accumulated equivalent creep strain in a single cycle reaching 0.0171. After three complete cycles, the total accumulated strain increased to 0.0362.

This phenomenon, where the accumulated strain in the initial cycle significantly exceeds that of subsequent cycles, is attributed to the initial stress redistribution and the transient viscoplastic response of the material. At the onset of the first thermal cycle, the solder joints undergo substantial inelastic flow to accommodate the sudden mismatch in thermal expansion among the package components from an initial stress-free state. Simultaneously, the internal state variable in the Anand model evolves rapidly to reach a dynamic equilibrium between strain hardening and recovery. As the cycling progresses (Cycle 2 and Cycle 3), the stress–strain hysteresis loop gradually stabilizes, and the creep strain accumulation transitions from a transient high-growth phase to a steady-state growth phase, resulting in a linear-like increase in total strain.

**Figure 18:**
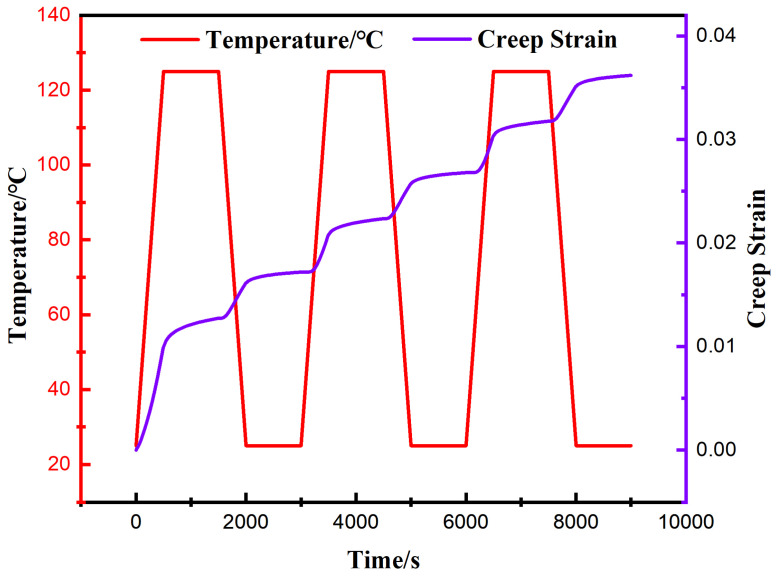
Dynamic correspondence between temperature cycling curves and equivalent creep strain response.

To provide an in-depth analysis of the correlation between creep behavior and temperature load, Figure 18 presents a comparative analysis of the temperature cycling profile and the creep strain evolution curve. The results indicate that the accumulation of creep strain is primarily concentrated during the temperature ramping stages (heating and cooling). In contrast, during the isothermal dwell stages, the creep curve exhibits distinct plateau characteristics, with minimal growth magnitude. This phenomenon strongly confirms that the difference in CTE among package components is the fundamental cause driving the deformation of solder joints. Specifically, during the ramping stages, the continuously changing temperature difference induces severe thermal mismatch displacement among components, thereby driving a rapid increase in creep strain. Conversely, during the dwell stages, due to the absence of additional thermal mismatch driving force, the creep accumulation rate significantly decreases and tends to stabilize.

Based on the aforementioned finite element analysis results, creep deformation has been confirmed as the dominant mechanism for accumulating damage and inducing failure in solder joints under thermal cycling loads [30,31]. Therefore, selecting a life prediction model capable of accurately reflecting the creep damage accumulation effect is crucial. Among existing reliability theories for lead-free solders, the Syed model is widely recognized for its ability to establish a precise power law relationship between the accumulated equivalent creep strain per cycle extracted from FE simulation and fatigue life. This model not only effectively circumvents the over-reliance on complex physical experimental data but has also been validated by extensive literature as applicable to the life assessment of various packaging structures and loading modes [32,33,34]. Accordingly, this study adopts the Syed model as the core criterion to quantitatively predict the thermal fatigue life of SAC0307-3Bi solder joints.

Given the current scarcity of literature regarding the fatigue ductility coefficients specific to the SAC0307-3Bi composition, and considering that this solder remains within the Sn-Ag-Cu alloy family, empirical constants for standard SAC solder were adopted as an approximate benchmark. This approach is further justified by the fact that the primary objective of this study is to reveal the evolutionary trends of solder joint life under various thermal cycling conditions, rather than to determine absolute numerical values. The general expression of the Syed life prediction model [32] is given as follows:

(11) $$N_{f}\;=\;{\left({C^{\prime }\varepsilon }_{acc}\right)}^{-1}$$

$N_{f}$ = Total number of cycles before failure;

$\varepsilon_{acc}$ = Creep strain accumulated in one cycle;

C’ = A constant representing the inverse of creep ductility.

The constant C’, corresponding to the inverse creep ductility of standard SAC series solders, is taken as 0.0468. Consequently, Equation (11) can be expressed as:

(12) $$N_{f}={\left({0.0468\varepsilon }_{acc}\right)}^{-1}$$

It is important to note that although the solid solution strengthening effect of Bi elements may exert some influence on the fracture ductility of the material, the suppression effect of Bi on creep strain accumulation has already been accounted for in the preceding simulation stage by modifying the constitutive model parameters. Consequently, the value of $\varepsilon_{acc}$ substituted into the equation inherently incorporates the impact of material modification, ensuring that the model remains effective in characterizing the relative fatigue reliability of the solder joints.

As illustrated in Figure 17, during the initial stage of thermal cycling loading, the creep response of the solder joints exhibits significant transient characteristics. It is not until the completion of the second cycle that the stress–strain behavior gradually achieves a stable hysteresis state. Given that the data from the first cycle are heavily influenced by initial stress redistribution and are thus not representative, they were excluded from the life prediction calculations.

To ensure computational accuracy, the accumulated equivalent creep strain increments from the stable second and third cycles were selected and calculated as an arithmetic mean, yielding a parameter value of $\varepsilon_{acc}=0.00954$. Substituting this value into Equation (12), the predicted creep–fatigue life of the lead-free solder joint is approximately 2239 cycles.

## 5. Conclusions

1. Determination of Constitutive Parameters: By combining the Norton creep equation with virtual tensile numerical simulations, the nine parameters (specifically A, Q/R, m, ξ, h_0_, a, $\hat{s}$, n and s_0_) of the Anand viscoplastic constitutive model for SAC0307–3Bi lead-free solder were successfully determined through inverse analysis. This establishes a reliable material modeling foundation for accurately simulating the nonlinear mechanical behavior of solder joints under complex thermo-mechanical coupling environments.
2. Identification and Quantification of Critical Failure Zones: Numerical analysis results clarified that the stress distribution within the solder array exhibits a significant dependence on the “Distance to Neutral Point (DNP).” The critical failure region was identified at the chip-side interface of the outermost corner solder joint, where the simulated peak von Mises equivalent stress reached 11.41 MPa. This finding confirms that the mismatch in coefficients of thermal expansion (CTE) between the silicon chip and the BT substrate is the dominant mechanical factor driving stress concentration and failure.
3. Creep Evolution Characteristics and Engineering Recommendations: The mechanical response of the solder joints demonstrates a strong temperature dependence. Equivalent creep strain accumulates primarily in a “step-wise” manner during the heating and cooling ramping stages. Conversely, during the initial phase of the low-temperature dwell, the solder joints retain high residual stress; due to the limited creep relaxation effect, stress levels cannot decrease rapidly. This finding holds significant engineering implications, suggesting that in practical applications, minimizing the duration and frequency of exposure to extreme low-temperature environments is crucial for mitigating stress damage and extending device service life.
4. Fatigue Life Prediction: Based on the steady-state accumulated equivalent creep strain per cycle $\left(\varepsilon_{acc}\right)$ extracted from the critical element of the corner solder joint, the thermal fatigue life of the SAC0307–3Bi solder joint was quantitatively assessed using the Syed model. The results indicate that under the investigated thermal cycling loading conditions, the predicted fatigue life of this lead-free solder joint is approximately 2239 cycles.

In terms of practical engineering application, the calibrated parameters facilitate cost-effective virtual prototyping, reducing reliance on expensive physical testing. Furthermore, the identified failure mechanisms suggest specific design optimizations, such as employing underfill reinforcement for critical corner joints and minimizing dwell times in extreme low-temperature environments to extend fatigue life.

## Figures and Tables

**Table 1:** Temperature and Strain rate of simulation.

Temperature/°C	Strain Rate 1/s^−1^	Strain Rate 2/s^−1^	Strain Rate 3/s^−1^	Strain Rate 4/s^−1^	Strain Rate 5/s^−1^
25	3 × 10^−5^	1 × 10^−4^	5 × 10^−4^	1 × 10^−3^	3 × 10^−3^
75	3 × 10^−5^	1 × 10^−4^	5 × 10^−4^	1 × 10^−3^	3 × 10^−3^
125	3 × 10^−5^	1 × 10^−4^	5 × 10^−4^	1 × 10^−3^	3 × 10^−3^

**Table 2:** Saturation stress at different temperatures and strain rates.

Temperature/°C	Strain Rate/s^−1^	Saturation Stress/MPa
25	3.0 × 10^−5^	28.66
	1.0 × 10^−4^	38.36
	5.0 × 10^−4^	54.43
	1.0 × 10^−3^	64.03
	3.0 × 10^−3^	84.02
75	3.0 × 10^−5^	12.41
	1.0 × 10^−4^	16.11
	5.0 × 10^−4^	23.59
	1.0 × 10^−3^	27.46
	3.0 × 10^−3^	32.35
125	3.0 × 10^−5^	6.33
	1.0 × 10^−4^	8.72
	5.0 × 10^−4^	12.46
	1.0 × 10^−3^	14.66
	3.0 × 10^−3^	18.96

**Table 3:** Anand model parameters.

Parameter Meaning	Anand Const.	Unit	Value
Initial value of deformation resistance	S_0_	MPa	26.09
Activation energy	Q/R	K	8530
Pre-exponential factor	A	1/s	224,585
Stress coefficient	ξ	-	4.68
Strain rate sensitivity	m	-	0.1832
Hardening constant	h_0_	MPa	8850
Coefficient for deformation resistance saturation value	$\hat{s}$	MPa	35.52
Strain rate sensitivity of deformation resistance saturation value	n	-	0.036
Strain rate sensitivity of hardening	a	-	1.33

**Table 4:** Dimensions of different components of the BGA [28].

Components	Dimensions (mm)
Solder joint dia.	0.46
Standoff Height	0.34
Solder joint pitch	0.60
Silicon die	7 × 7 × 0.28
BT substrate	10 × 10 × 0.42
Mold compound	10 × 10 × 0.47
PCB substrate	15 × 15 × 0.57

**Table 5:** Material properties of BGA package elements [3].

BGA Element	Elastic Modulus (E), GPa	Poisson’s Ratio, (ν)	CTE (×10^−6^), K^−1^
Solder joint	50	0.3	20
Silicon die	131	0.30	2.8
BT substrate	18.2	0.25	15.0
Mold compound	15.5	0.25	15.0
PCB substrate	22	0.28	18

## Data Availability

The original contributions presented in this study are included in the article. Further inquiries can be directed to the corresponding authors.

## Abbreviations

**Abbreviation**	**Full Term**
BGA	Ball Grid Array
CSP	Chip Scale Package
CTE	Coefficient of Thermal Expansion
DNP	Distance to Neutral Point
EMC	Epoxy Molding Compound
FC	Flip Chip
FEA	Finite Element Analysis
IMC	Intermetallic Compound
PCB	Printed Circuit Board
SAC	Sn-Ag-Cu (Tin–Silver–Copper)
UTS	Ultimate Tensile Strength
